# Transhiatal bilateral thoracic duct ligation for duplicated thoracic duct injury after esophagectomy: a case report

**DOI:** 10.1186/s40792-022-01567-7

**Published:** 2022-12-02

**Authors:** Shuhei Komatsuzaki, Katsuji Hisakura, Koichi Ogawa, Yoshimasa Akashi, Jaejeong Kim, Shoko Moue, Yoshihiro Miyazaki, Kinji Furuya, Manami Doi, Yohei Owada, Osamu Shimomura, Yusuke Ohara, Kazuhiro Takahashi, Shinji Hashimoto, Tsuyoshi Enomoto, Naoto Koike, Tatsuya Oda

**Affiliations:** 1 Department of Gastrointestinal and Hepato-Biliary-Pancreatic Surgery, University of Tsukuba, 1-1-1 Tennoudai, Tsukuba, Ibaraki 305-8575 Japan; 2 Department of Surgery, Seirei Sakura Citizen Hospital, 2-36-2 Eharadai, Sakura, Chiba 285-8765 Japan; 3 Department of Surgery, Hitachi, Ltd., Hitachinaka General Hospital, 20-1, Ishikawacho, Hitachinaka, Ibaraki 312-0057 Japan

**Keywords:** Chylothorax, Esophagectomy, Transhiatal, Left thoracic duct, Thoracic duct ligation

## Abstract

**Background:**

The treatment of duplicated thoracic ducts (TDs) injury after esophagectomy generally requires a bilateral transthoracic approach. We present the cases of two patients with postoperative chylothorax who underwent transhiatal bilateral TD ligation for duplicated TDs.

**Case presentation:**

Two patients diagnosed with chylothorax after esophagectomy performed for thoracic esophageal cancer underwent transhiatal TD ligation. Although supradiaphragmatic mass ligation was performed on the fat tissue of the right side of the aorta containing the TD, chyle leakage persisted. To tackle this, the fat tissue of the left side of the aorta was ligated, after which the chyle leakage stopped.

**Conclusion:**

Compared to the conventional transthoracic approach, the transhiatal approach enables the ligation of both left- and right-sided TD in a single surgical operation, without the need to change the patient’s posture. This approach may be appropriate for the treatment of chylothorax after esophagectomy, considering the possibility of duplicated TDs.

## Background

Chylothorax is a rare but troubling complication of esophagectomy because it leads to hypovolemia, malnutrition, and immunosuppression [1]. The incidence of chylothorax is 1.1–3.8% [2–7]. Conservative management with intrapleural drainage, total parenteral nutrition, and octreotide usually takes several weeks to provide any improvement and is frequently unsuccessful; therefore, surgical treatment is eventually required [2].

In general, the thoracic duct (TD) runs along the right side of the aorta, crosses the left side in the upper mediastinum, and finally flows into the left venous angle. However, major configuration variations are noted in 14% of patients; in particular, 7.8–10.6% of patients have a left-sided TD [8–10]. A left-sided TD injury cannot be repaired with the right transthoracic approach; therefore, left-sided TD injuries are commonly treated via the left transthoracic approach [11–14].

On the other hand, the transhiatal approach is simple, easy to perform, and preferable for patients who should avoid transthoracic procedures because of severe intrathoracic adhesions or cardiopulmonary problems [15, 16]. Moreover, the TD can theoretically be ligated with this approach on the left and right sides of the aorta in the supradiaphragmatic space, conferring extra versatility to this approach. We present the cases of two patients with postoperative chylothorax who underwent transhiatal bilateral TD ligation for duplicated TDs.

## Case presentation

### Patient 1

**Fig. 1:**
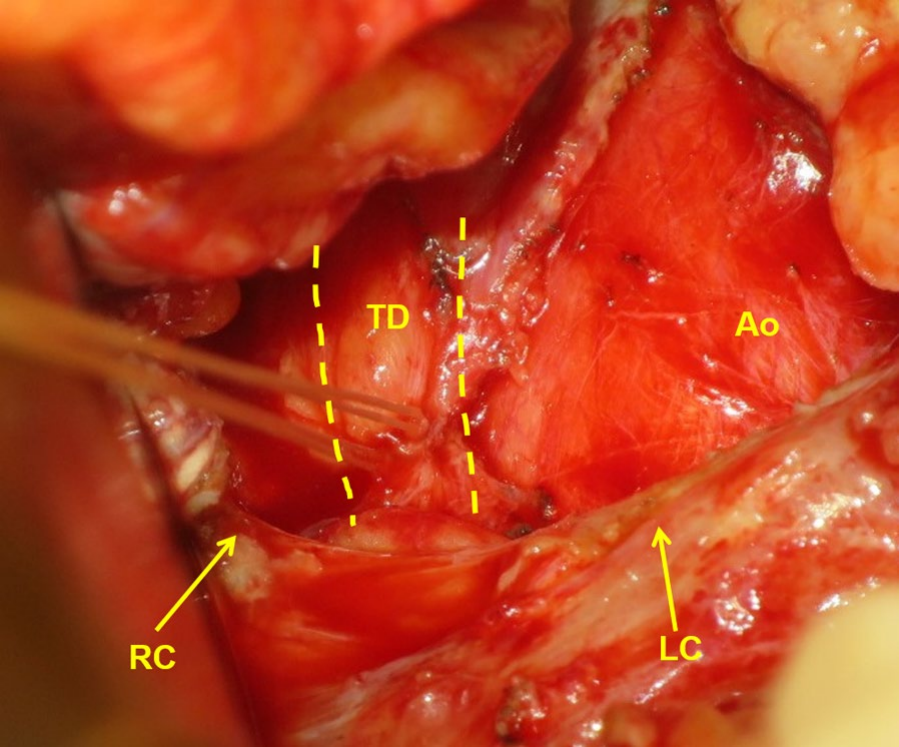
Ligation of a right-sided thoracic duct. Mass ligation of the fat tissue surrounded by the right wall of the aorta, the azygos vein, and the vertebral body, which contains the right-sided thoracic duct. *TD* thoracic duct, *RC* right crus of the diaphragm, *LC* left crus of the diaphragm, *Ao* aorta

**Fig. 2:**
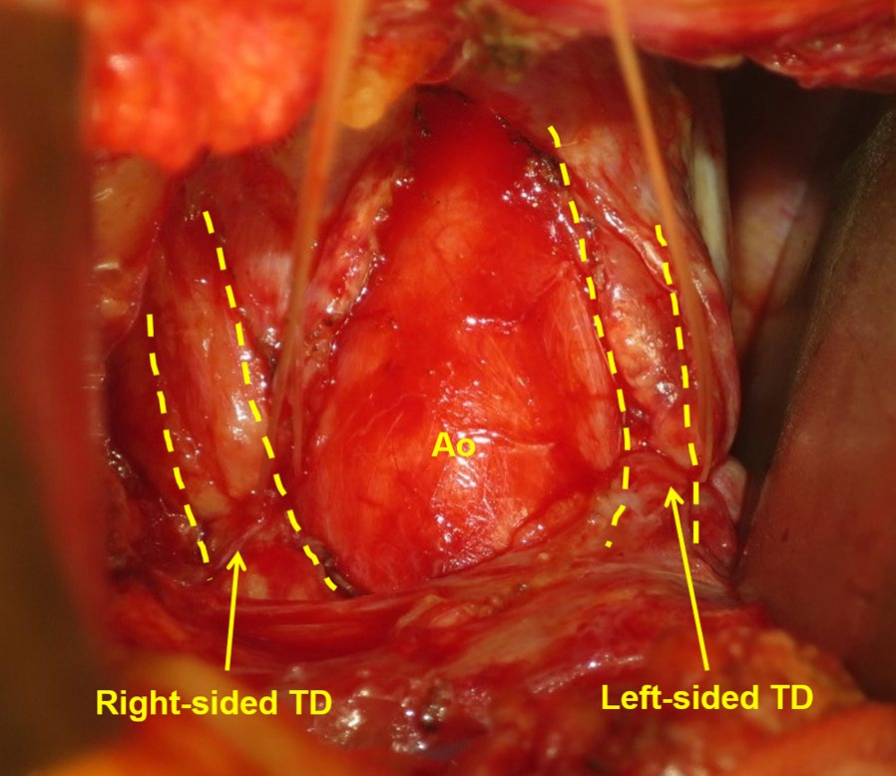
Ligation of a left-sided thoracic duct. Mass ligation of the fat tissue on the left side of the aorta which contains the left-sided thoracic duct. *TD* thoracic duct, *Ao* aorta

**Fig. 3:**
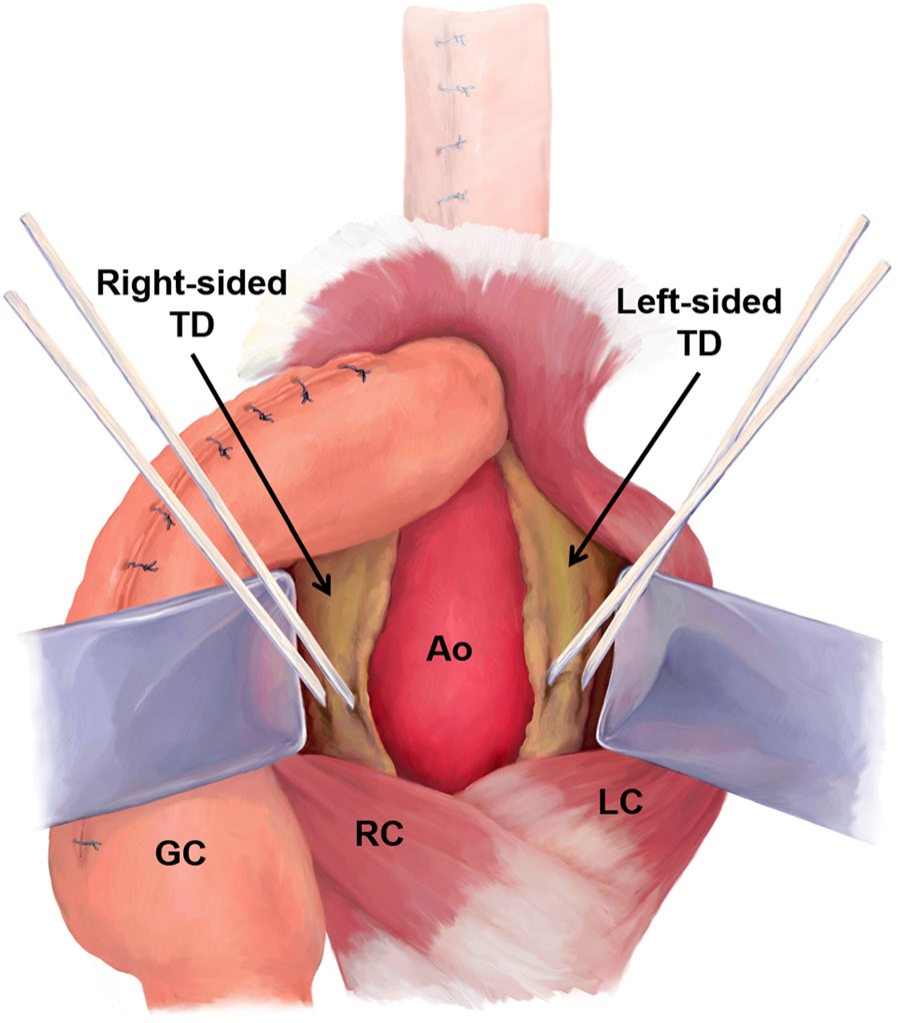
Illustration of the thoracic duct ligation. *TD* thoracic duct, *RC* right crus of the diaphragm, *LC* left crus of the diaphragm, *Ao* aorta, *GC* gastric conduit

A 54-year-old man with clinical T1bN1 upper thoracic esophageal carcinoma underwent neoadjuvant chemotherapy, followed by radical esophagectomy with gastric conduit reconstruction via the posterior mediastinal route. The TD was preserved. On the 2nd day after surgery, a large amount of chest drainage, which had increased to 2000 mL or more, was diagnosed as chylothorax. Conservative treatment with fasting and total parenteral nutrition was immediately initiated; however, the drain output did not reduce. Additionally, octreotide was administrated, without effects. Because no radiologist was available to perform lymphangiography, surgical treatment (TD ligation) was suggested, and the patient consented on day 14 after esophagectomy. Because duplicated TD has been reported previously, we routinely choose the transhiatal approach [8–10, 17]. To visualize the chyle leakage, whole milk was administered through the nasojejunal tube preoperatively. The lower posterior mediastinum was re-explored through laparotomy and phrenotomy. After gently displacing the gastric conduit, the site of chyle leakage could not be identified. Mass ligation was performed in the supradiaphragmatic space on the fat tissue surrounded by the right wall of the aorta, azygos vein, and vertebral body (Fig. 1). However, the chyle leakage from the chest drain persisted. To combat this, mass ligation was performed on the fat tissue on the left side of the aorta (Figs. 2, 3), after which the drainage volume decreased rapidly and the turbidity of the lavage fluid disappeared. The chyle leakage was determined to have stopped. The operation time was 139 min and the blood loss was 270 mL. Liquid diet was initiated on the 3rd day after the repeat surgery and the subsequent restoration of solid food intake was uneventful; the amount of chest drainage did not increase, and the chest drainage tube was removed on day 7. The patient was discharged on day 12, with no pleural effusion on radiography.

### Patient 2

A 58-year-old man with clinical T1bN0 middle thoracic esophageal cancer underwent radical esophagectomy with gastric conduit elevation via the posterior mediastinal pathway in which the TD was preserved. On the 9th day after the first surgery, the amount of chest drainage increased to more than 2000 mL, leading to a diagnosis of chylothorax. Conservative treatment with fasting, total parenteral nutrition, and octreotide was not effective. The patient was reluctant to undergo surgery at first; however, he eventually consented. On the 28th day, transhiatal TD ligation was performed. The same procedure as that for the first patient was performed; mass ligation had to be similarly performed on the fat tissue on the left side of the aorta to stop chyle leakage. The operation time was 133 min and the blood loss was 55 mL. Oral intake was initiated on the 3rd day after the second surgery. Chest drainage did not increase, and the chest drainage tube was removed on day 5, with no accumulation of pleural effusion.

## Discussion

We have reported two cases of chylothorax after radical esophagectomy who underwent surgical intervention using a transhiatal approach, Both patients were successfully treated via supradiaphragmatic TD ligation on both sides of the descending aorta in a single surgical site during a single-stage surgery for duplicated TDs. Transhiatal TD ligation did not require one-lung ventilation and could be performed without direct visualization of the injured intrathoracic area. To our knowledge, this is the first report of bilateral TD ligation performed with the transhiatal approach for chylothorax after esophagectomy.

Left-sided TD has been previously described. In 1953, Adachi et al. performed 261 autopsies and described nine types of anatomical variation [17]. Types I, II, and III involve bilateral TDs; types IV, V, and VI involve right-sided TD; and types VII, VIII, and IX involve left-sided TD. Furthermore, types I, IV, and VII involve bilateral outflow; types II, V, and VIII involve right outflow; and types III, VI, and IX involve left outflow. None of the autopsies revealed type I, II, VII, and VIII TDs. Therefore, in clinical practice, patients with left-sided TD are classified as Type III or IX, and the frequency of left-sided TD is as high as 7.8–10.6% [8–10]. Transthoracic ligation of the TD is the standard surgical procedure for post-esophagectomy chylothorax; therefore, bilateral TDs are commonly treated with a bilateral transthoracic approach [3, 11–14]. This approach requires changing the patients’ posture during surgery, extending the surgical duration. Conversely, the transhiatal approach allows the ligation of right and left-sided TDs in a single surgical site and during a single-stage surgery in the supine position.

Transhiatal TD ligation in the supradiaphragmatic space was first described by Miyamura et al. [15]. Suzuki et al. later reported it as an effective surgical treatment for chylothorax [16]. The lower posterior mediastinum was released for re-exploration through phrenotomy. The left-sided TD could be ligated using a procedure similar to that used for the right side in a single surgical site. The ability to ligate bilateral TDs through a hiatal approach is the major advantage of this approach in treating TD injury. Other studies have described that the retraction of the conduit and adhesiolysis are not difficult because this space has typically been dissected during the initial surgery and is friable [18]. Even in our patients, the surgical site was easily exposed without adhesiolysis.

Another advantage of the transhiatal approach is the minimal cardiopulmonary invasiveness in which one-lung ventilation is avoided [16, 19, 20]. While patients with postoperative chylothorax have undergone major thoracic surgery and are often unsuitable for a transthoracic approach because of debilitation, the transhiatal approach minimizes the impact on circulatory and respiratory dynamics. Moreover, the identification and treatment of the actual site of the injured TD are not necessary with this approach [15, 16, 21]. The TD tends to be injured around the tracheal bifurcation during esophagectomy; however, due to the preceding surgery, the actual site of injury is difficult to detect intraoperatively [20, 22]. In the transhiatal approach, the TD is ligated at the supradiaphragmatic space (the afferent side of the injured lesion); thus, the lymph flow to the damaged area can be blocked [4, 15, 16]. Owing to these advantages, the transhiatal procedure is useful for patients who are ineligible for a transthoracic approach; for example, those who have undergone pulmonary surgery and esophagectomy.

The treatment for chylothorax remains controversial and evidence for the optimal management is lacking because of its low incidence. Radiological procedures using imaging devices, such as percutaneous lymphangiography, are superior in terms of reducing mortality; however, their effectiveness is presumably lower than that of surgery [7]. Recently, the transhiatal laparoscopic approach was reported as a safe and fast procedure for the treatment of chylothorax [19, 23–26]. The laparoscopic approach was not adopted in our patients because it prioritizes gentle manipulation of the gastric conduit. This approach may be adopted based on proficiency.

## Conclusions

Transhiatal TD ligation is a feasible option, especially for patients with a potential for duplicated TDs. However, no study before ours has contributed to the treatment of left-sided TD injury through the hiatus.

## Data Availability

Not applicable.

## Abbreviation

TD: Thoracic duct
